# Targeting cellular senescence through nutritional senotherapeutics in brain aging and neurodegenerative diseases

**DOI:** 10.3389/fnut.2026.1824908

**Published:** 2026-08-03

**Authors:** Verónica Salas-Venegas, Norma E. López-Diazguerrero, Ricardo J. Ramírez-Carreto, Mina Konigsberg, Anahí Chavarría

**Affiliations:** 1 Unidad de Investigación en Medicina Experimental, Facultad de Medicina, Universidad Nacional Autónoma de México, CDMX, Mexico City, Mexico; 2 Departamento de Ciencias de la Salud, División de Ciencias Biológicas y de la Salud (DCBS), Universidad Autónoma Metropolitana Iztapalapa, CDMX, Mexico City, Mexico; 3 Programa de Doctorado en Ciencias Biomédicas, Universidad Nacional Autónoma de México, CDMX, Mexico City, Mexico

**Keywords:** brain, neurodegenerative disease, nutritional senotherapeutic, senescence, senolytic, senomorphic

## Abstract

Cellular senescence has emerged as a key contributor to brain aging and neurodegenerative diseases, which are increasing in prevalence as life expectancy rises. Senescent cells accumulate in multiple brain cell populations and secrete the senescence-associated secretory phenotype (SASP), promoting chronic neuroinflammation, mitochondrial dysfunction, blood–brain barrier disruption, and progressive neuronal damage. This review examines the role of cellular senescence in brain aging and neurodegenerative diseases and discusses senotherapeutic strategies aimed at either eliminating senescent cells (senolytics) or modulating the SASP (senomorphics). Particular emphasis is placed on nutritional senotherapeutics, naturally occurring bioactive compounds derived from dietary sources that target molecular pathways involved in cellular senescence. Senomorphic compounds such as sulforaphane, curcumin, and resveratrol primarily attenuate oxidative stress and inflammatory signaling, whereas senolytic agents including quercetin and fisetin selectively promote the elimination of senescent cells by disrupting pro-survival pathways. Current evidence from experimental models and emerging clinical studies suggests that nutritional senotherapeutics represent promising complementary strategies for modulating cellular senescence and promoting brain health. However, their clinical translation remains constrained by limited human evidence, low bioavailability, and unresolved challenges related to optimal dosing and blood–brain barrier penetration, highlighting the need for well-designed clinical trials before their therapeutic potential can be fully established.

## 1 Introduction

The rise in the elderly population has brought an increase in the prevalence of age-related neurodegenerative diseases, including Alzheimer’s (AD) and Parkinson’s (PD) diseases, resulting in substantial morbidity, mortality, and considerable healthcare costs (1, 2). One of the pathological signatures of neurodegenerative diseases is the accumulation of misfolded proteins into non-degradable oligomers, which drive cytotoxic and inflammatory processes and ultimately lead to synaptic impairment and neuronal death (3); however, the complete pathogenesis of these diseases remains poorly understood.

Recent evidence indicates that cellular senescence is a key contributor to neurodegenerative disease pathogenesis (4). Cellular senescence occurs as a result of exhausted replication or in response to harmful stimuli, such as increased oxidative stress and sustained DNA damage. Nevertheless, the most significant feature of senescent cells is the secretion of a complex array of pro-inflammatory factors, collectively referred to as the senescence-associated secretory phenotype (SASP) (5).

Markers of cellular senescence have been identified in different brain cell types (neurons, astrocytes, microglia, etc.) in older adults, as well as in *in vitro* and *in vivo* models of neurodegenerative diseases (3, 6). In recent years, multiple therapeutic approaches to eliminate senescent cells or modulate pro-inflammatory factors in the SASP have received significant attention (2).

Compared with conventional pharmacological senotherapeutics, nutritional senotherapeutics should be regarded as complementary rather than replacement strategies for promoting healthy aging. Among the various senotherapeutic strategies currently under investigation, dietary bioactive compounds have emerged as promising complementary approaches for promoting healthy aging. Their natural occurrence in commonly consumed foods, relative affordability, broad accessibility, and potential suitability for long-term dietary interventions make them attractive candidates for preventive strategies to delay the accumulation of senescent cells and reduce chronic low-grade inflammation (7, 8). Despite these potential advantages, their clinical translation remains limited by challenges, including low bioavailability, uncertainty about optimal dosing, and the need for robust clinical trials to establish their efficacy in neurodegenerative diseases. Therefore, nutritional senotherapeutics are best viewed as complementary components of preventive and precision-based geroscience strategies rather than stand-alone therapeutic alternatives.

Here, we aim to discuss the role of cellular senescence in the progression and pathogenesis of neurodegenerative diseases, as well as the therapeutic strategies targeting senescence from dietary sources that influence metabolic pathways critical for neuronal health, and outline the significant challenges in eliminating or modulating this complex phenomenon.

## 2 Aging brain and senescence

Aging is a complex phenomenon characterized by a progressive loss of physiological integrity, leading to functional decline and increased vulnerability to mortality in organisms. Twelve distinctive hallmarks have been proposed to be present during the aging process: cellular senescence, telomere attrition, epigenetic alterations, genomic instability, loss of proteostasis, stem cell exhaustion, deregulated nutrient sensing, mitochondrial dysfunction, impaired macroautophagy, altered intracellular communication, chronic inflammation, and dysbiosis (9).

The brain is no exception; as with other organs and systems, its functional capacities progressively decline with age. Among the most detrimental effects on daily life are reduced cognitive performance, including learning and memory, attention, sensory perception (including hearing, vision, touch, taste, and smell), motor coordination, and decision-making speed (10).

In recent years, markers of cellular senescence have been identified in different types of brain cells. Accumulation of senescent cells in the brains of older adults has been implicated in the onset and progression of neurodegenerative diseases, such as PD, and in cognitive decline (3, 6).

### 2.1 Senescence

Cellular senescence is a phenomenon characterized by the arrest of cell cycle progression (11, 12). Different stimuli, both external and internal, can trigger cellular responses and senescence states. Most of these stimuli, including oxidative stress, neuroinflammation, high-fat diets, elevated glucose levels, and proteostasis disruption, among others, induce DNA damage (13, 14), which activates various signaling pathways, such as p53, p16/Rb, and p21 (15). These pathways are involved in cell cycle arrest (12).

Senescent cells exhibit distinct biochemical and morphological features that have been used as markers for their detection in cell cultures or tissues. Differences are reported between species, cell types, and even at the individual cell level, resulting in differential activation of senescence-mediating pathways, which makes it challenging to find a specific marker for this phenomenon (16). Some of the most common markers include overexpression of CDK inhibitors that prevent the phosphorylation of CDKs, telomere attrition, loss of lamin B1 expression, DNA damage scars, increased activity of the senescence-associated *β*-galactosidase enzyme (SA-β-gal), dysfunctional mitochondria with augmented reactive oxygen species (ROS) production, macromolecular aggregate accumulation, and increased secretory SASP activity (17). The components of the SASP are heterogeneous and dynamic; factors that can generate this variation include cell type and senescence-inducing agents. The components can be divided into two main categories: soluble signaling factors (growth factors, chemokines, and interleukins), proteases, and insoluble proteins/extracellular matrix (ECM) components (18).

Cellular senescence plays physiological roles in tissue homeostasis, repair, and tumor suppression (19); however, pathological accumulation of these cells in aging tissues and age-related conditions has been associated with tissue deterioration and disease. Thus, senescence represents a cellular program with dual effects, consistent with the concept of antagonistic pleiotropy, whereby its effects depend on the time cells remain in the tissues (11).

Moreover, it is possible to consider their key role in the development and progression of different pathologies. Senescent cells, including neurons and glial cells, have been found in the CNS of patients and animals (Table 1) (3, 6). In fact, the accumulation of senescent cells in brain cell populations has expanded our understanding of its impact on brain aging (5, 9).

**Table 1:** Different brain cell types, physiological functions, and senescence features.

Brain cells	Physiological function	During senescence	Reference
Neurons	Electrically excitable cells that transmit signals, employing both electrical and chemical components in the transmission of information.	Altered communication through changes in response to electrical stimulation, including spontaneous firing rates and excitability. SASP secreted by neurons may contain molecules that affect neurotransmitter release and activity, as evidenced in a study of neuronal cell cultures, which observed increased α-synuclein secretion in response to senescent stimuli.	(40, 106)
Microglia	Resident macrophages in the CNS. Maintenance of brain homeostasis by regulating neuronal activity and phagocytosing cellular debris. Immunosurveillance through mechanisms such as chemotaxis, phagocytosis, antigen presentation, and cytokine production. Dysregulated functions are associated with inflammatory responses and the pathogenesis of neurodegenerative diseases.	Show morphological debranching, reduced ability to proliferate, increased cell body hypertrophy, and release of the SASP. Display markers of cellular senescence: increased SA-*β*-gal activity, mitochondrial dysfunction, and elevated oxidative stress. A link between microglial senescence and the development of diseases has been established, which contributes to brain deterioration during aging and in neurodegenerative diseases, such as AD and PD.	(12, 22, 107, 108)
Astrocytes	The most abundant glial cell type in the brain. They are responsible for complex and essential functions in CNS physiology, including nutrient delivery to neurons, regulation of synaptic plasticity, Ca^2+^-dependent neurotransmitter release, support of the blood–brain barrier (BBB), and maintenance of extracellular ionic balance.	Studies using human brain samples have shown an increase in senescent astrocytes with age. Various animal models have demonstrated a relationship between glial cell senescence and neurodegenerative processes, characterized by loss of function and secretion of SASP factors. *In vitro* studies have revealed marked impairments in essential functions, including phagocytic activity, tissue regenerative capacity, metabolism, and neuroprotective roles. The SASP of senescent astrocytes alters neuronal mitochondrial function.	(27, 34, 109, 110)
Other cell types (oligodendrocytes, endothelial cells, and pericytes)	Oligodendrocytes produce myelin sheaths to ensure the saltatory propagation of action potentials along axons. Endothelial cells line the blood vessel lumen and restrict paracellular flux through tight junctions, thereby forming a physical barrier that prevents substances from passing across the vessel wall. Pericytes coordinate many functions, including angiogenesis, BBB formation and maintenance, vascular stability and angioarchitecture, regulation of capillary blood flow, and clearance of toxic cellular byproducts, all of which are necessary for proper CNS homeostasis and neuronal function.	The senescence of these different cell types can impair myelination and alter BBB permeability, allowing the entry of unwanted factors and leading to loss of brain function. Senescent pericytes can compromise BBB integrity by interacting with glial cells. Pericytes are implicated in many age-related diseases. For example, degeneration of gray matter pericytes has been linked to diseases characterized by cognitive deterioration, including AD and other dementias.	(6, 111–114)

Cells in the CNS exhibit senescence-related characteristics that affect their function.

Recent evidence indicates that cellular senescence is highly cell-type-dependent within the central nervous system, with distinct brain cell populations exhibiting unique senescence-associated phenotypes and functional consequences. In neurons, senescence-like features are characterized by persistent DNA damage responses, altered proteostasis, and the secretion of pro-inflammatory mediators despite their post-mitotic nature (20). Senescent astrocytes exhibit an enhanced SASP, impaired metabolic and trophic support, and reduced capacity to maintain neuronal homeostasis, thereby exacerbating neuroinflammation (21). Senescent microglia display chronic inflammatory activation, defective phagocytosis, and sustained cytokine secretion, which contribute to synaptic dysfunction and neuronal injury (22). In oligodendrocyte progenitor cells, senescence limits remyelination and tissue repair, whereas endothelial cell senescence compromises blood–brain barrier (BBB) integrity and cerebral vascular homeostasis (23, 24). These findings indicate that cellular senescence is heterogeneous in the brain, suggesting that effective senotherapeutic strategies may require cell type-specific approaches rather than uniformly targeting all senescent cell populations.

In the brain, these nondividing cells accelerate biological aging by losing the ability to participate in repair and regeneration processes (24), and the SASP can also transmit and reinforce the senescent state phenotype via both autocrine and paracrine mechanisms (25). The accumulation of senescent cells, resulting from the evasion of immune-mediated clearance in the aged brain, may contribute to cognitive decline by promoting neuroinflammation (26).

Hence, cellular senescence has emerged as a critical mechanism that mediates the brain cellular dynamics driving neurodegenerative progression (5). In addition to its pro-inflammatory effects, the SASP may contribute to neurodegenerative processes by altering and disrupting cell–cell contacts at the BBB, which are essential for proper neuron–glia interactions and maintaining brain homeostasis (14, 27).

The use of cellular senescence markers in humans is limited by tissue accessibility, cellular heterogeneity, and the absence of a universal senescence-specific biomarker (28, 29). Consequently, increasing attention has been directed toward emerging approaches that combine multiple indicators of biological aging. Circulating SASP components, including IL-6, IL-1β, TNF-*α*, GDF15, CCL2, and matrix metalloproteinases, have shown promise as minimally invasive biomarkers reflecting systemic senescent cell burden, although their specificity remains limited because they are also elevated in other inflammatory conditions (30, 31). Moreover, transcriptomic signatures derived from senescence-associated gene expression profiles are emerging as valuable tools for assessing biological age and senescence-related molecular alterations (32). Collectively, these approaches suggest that integrating circulating biomarkers with molecular and omics-based signatures may provide a more reliable strategy for monitoring senescence and evaluating the efficacy of senotherapeutic interventions in clinical settings.

#### 2.1.1 Senescence in neurodegenerative diseases

Cellular senescence is implicated in the etiopathogenesis of AD and PD, as these disorders share several mechanisms that promote senescence and induce or exacerbate neurodegeneration.

The aberrant accumulation of senescent cells ultimately disrupts organ morphology and impairs physiological function (25). In particular, cellular senescence can disrupt proteomic function and homeostasis, altering normal rates of protein synthesis and degradation, and producing misfolded proteins or abnormal protein aggregates; these, in turn, can contribute to the development and progression of AD and PD (33).

##### 2.1.1.1 Alzheimer’s disease

AD is characterized by brain atrophy, evidenced by a progressive deterioration of memory and cognition. During this disease, extracellular accumulation of beta-amyloid peptide (Aβ) and neurofibrillary tangles (NFT) composed of tau protein, synaptic loss, and persistent neuroinflammation are present (27).

Several studies have demonstrated the involvement of senescence in AD; Zhang et al. (2019) reported that in the brains of post-mortem patients and mouse models of AD, oligodendrocyte progenitor cells exhibited a senescence-like phenotype, characterized by upregulation of p21 and p16, as well as increased SA-*β*-gal activity (34).

Musi et al. showed that NFT-bearing neurons from post-mortem AD brain tissue display a senescence-like transcriptomic profile, as revealed by increased expression of cell survival and inflammation genes, and lower expression of apoptosis-related genes (4). This senescence further promotes Aβ deposition and tau hyperphosphorylation, thus creating a self-reinforcing loop with AD pathology (35).

Building on this observation, SASP factors are detected in the blood and cerebrospinal fluid (CSF) of AD patients (IL-6, IL-9, IL-1α, IL-1β, MMP2, MMP9, GM-CSF). However, many of the SASP factors measured co-occur with multiple inflammatory responses, underscoring the need for continued biomarker discovery with greater specificity for senescence (36).

##### 2.1.1.2 Parkinson’s disease

PD is a neurodegenerative disease characterized by loss of motor function. The loss of dopaminergic neurons in the substantia nigra, along with the presence of Lewy bodies (cytoplasmic protein aggregates that contain a variety of proteins, including *α*-synuclein and ubiquitin), are the primary characteristics of PD (33). The risk of PD increases with age, male sex, and exposure to chemicals, such as pesticides (37).

Several studies have demonstrated that senescence is involved in the progression of PD. Raised p16 levels are present in autopsy tissue from the substantia nigra pars compacta (SNpc) of PD patients compared to age-matched patients without this pathology (38). Notably, levels of SASP factors, including MMP-3, IL-6, IL-1*α*, and IL-8, are elevated. Additionally, GFAP-positive astrocytes in SNpc exhibit reduced expression of the nuclear envelope protein lamin B1, a hallmark of cellular senescence (39).

Also, higher α-synuclein content in neurons with a senescent phenotype is present in *in vitro* assays (40). Neuroinflammation is induced by glial cell-derived pro-inflammatory SASP, particularly the secretion of IL-6, IL-8, and MMP3. The SASP secretion could explain the development of local inflammation, triggering a massive immune response and ultimately leading to the elimination of dopaminergic neurons (40).

In addition, SASP factors released by senescent microglia and astrocytes can induce senescence in dopaminergic neurons, leading to neuronal death. SASP-driven chronic inflammation leads to endothelial contraction and gap formation, facilitating T-cell infiltration into the brain (3). Infiltrating T cells then release chemokines and cytokines that promote dopaminergic neuron loss. In PD, glial cells and dopaminergic neurons exchange *α*–synuclein–containing exosomes, driving Lewy body formation (41).

The accumulation of senescent cells in the brain during aging and in neurodegenerative diseases emerges as a pivotal player in the etiopathological processes driving these disorders, through proteostasis disruption, altered protein synthesis and degradation, which favors the aggregation of misfolded proteins (3). It also compromises brain tissue regeneration and function, while SASP-mediated inflammation exacerbates neurodegenerative processes (33).

The evidence presented above underscores that the accumulation of senescent cells across multiple neuronal types is not simply a passive consequence of neurodegeneration but a potent driver of its progression. This understanding has shifted the therapeutic paradigm away from concentrating exclusively on the consequences of neurodegeneration, such as protein aggregates or synaptic loss. Emerging strategies now seek to address senescence itself as a pre-existing pathological node. In this context, senotherapy has emerged as a promising framework for intervening at the cellular level, either by eliminating senescent cells or by regulating their harmful secretory profile.

Although AD and PD remain the most extensively investigated neurodegenerative disorders in the context of cellular senescence, emerging evidence suggests that senescence-related mechanisms are also involved in other neurological diseases. In amyotrophic lateral sclerosis (ALS), senescence markers have been identified in microglia, and these changes have been associated with neuroinflammation and motor neuron loss (42, 43). In frontotemporal dementia (FTD), senescence-related pathways may intersect with tau- and TDP-43–associated proteotoxic stress, persistent DNA damage responses, and neuroinflammatory signaling (44, 45). In Huntington disease (HD), mutant huntingtin toxicity has been linked to cellular aging mechanisms, oxidative stress, mitochondrial dysfunction, and senescence-like phenotypes in neural cells (46, 47). Vascular cognitive impairment and vascular dementia are also closely related to cerebrovascular aging, where endothelial cell senescence may contribute to BBB dysfunction, vascular inflammation, and cognitive decline (48, 49). Although the evidence for these disorders is less extensive than for AD and PD, these findings support cellular senescence as a shared pathological mechanism across multiple neurodegenerative conditions.

## 3 Targeting cellular senescence: senotherapeutics

The clear relationship between the accumulation of senescent cells and neurodegenerative diseases has led to the development of therapies aimed at preventing this phenomenon using pharmacological compounds that inhibit it.

Senotherapy is a therapeutic approach that targets and manages senescent cells in the body. This approach can be categorized into two primary groups of senotherapeutics: *senolytics,* which selectively eliminate senescent cells, and *senomorphics*, which inhibit or modulate SASP-induced inflammation (12, 50). Most of these senotherapeutic compounds have been identified through pharmacological repositioning, a method that uses existing drugs for the treatment of a disease, rather than their original indication (51).

Senolytics are small molecules that induce apoptosis specifically in senescent cells by inhibiting anti-apoptotic signaling pathways. Senescent cells escape apoptosis by increasing anti-apoptotic pathways, commonly referred to as senescent cell anti-apoptotic pathways (SCAPs), which distinguishes them from healthy cells. Senolytic agents target this vulnerability by inhibiting these pro-survival mechanisms, selectively inducing apoptosis through regulation of apoptosis resistance (BCL-2, p53, and PI3K/AKT), while leaving non-senescent tissue largely unaffected (52).

On the other hand, molecules with senomorphic activity can limit the detrimental effects of senescence by attenuating, suppressing, or modulating specific components of SASP without causing cell death (16). The primary strategy of potential senomorphic therapies is to modulate the pathways implicated in SASP synthesis and release. Some of these pathways include p38 MAPK, JAK/STAT, and mTOR, as well as transcription factors such as NF-κB, C/EBPβ, and STAT3 (53).

Some of the most extensively studied molecules with senolytic and senomorphic activity, along with their mechanisms of action and natural or synthetic origin, are presented in Table 2.

**Table 2:** Senotherapeutic molecules: origin and mechanism of action.

**Origin**	**Molecule**	**Mechanism of Action**	**Reference**
**Senolytics of Synthetic origin**	Dasatinib	Tyrosine kinase inhibitor that upregulates senescent cell anti-apoptotic pathways (SCAPs). This drug is used to treat chronic myeloid leukemia and acute lymphoblastic leukemia.	(115)
	Navitoclax	A chemotherapeutic drug that targets Bcl-2 family members, including BCL-2, BCL-XL, and BCL-W.	(116)
	ABT-737	Selective small molecule B-cell lymphoma 2 (Bcl-2) Homology 3 (BH3) mimetic, with potential pro-apoptotic and antineoplastic activities. BCL-2 family inhibitors (Inhibit BCL-2, BCL-XL, BCL-W).	(117)
	Geldanamycin	A benzoquinone ansamycin antibiotic that manifests anti-cancer effects. The mechanism of action involves inhibiting the HSP90 Chaperone function.	(118)
	Digoxin	A cardiac glycoside that inhibits Na+/K+-ATPase upregulates BCL2-family protein NOXA.	(119)
**Senolytics of natural origin**	Quercetin	A flavonoid present in broccoli, tomatoes, onions, and celery that upregulates senescent cell anti-apoptotic pathways (SCAPs).	(93,94)
	Fisetin	A flavonoid present in many fruits and vegetables, such as apples, persimmons, grapes, onions, cucumbers, and strawberries. It regulates BCL-2, PI3K/AKT, and NF-κB pathways.	(66)
	Piperlongumine	A compound isolated from a variety of species in the genus Piper. It regulates OXR1 and NF-κB, and the pathways involved in these processes remain unclear.	(120)
**Senomorphics of Synthetic origin**	Metformin	Biguanide that modulates NF-κB, Nrf2, MBNL1, STAT3, AMPK, SIRT1, insulin/IGF-1, mTOR, and some other pathways that are unclear.	(121)
	Aspirin	A non-steroidal anti-inflammatory drug inhibits senescence-associated β-galactosidase activity and increases telomerase activity.	(122)
	Atorvastatin	A statin that inhibits HMG-CoA reductase and mediates activation of Akt.	(108)
**Senomorphics of natural origin**	Sulforaphane	Isothiocyanate is present in cruciferous vegetables. SFN activates antioxidant and anti-inflammatory responses by inducing Nrf2 and inhibiting NF-κB.	(76)
	Curcumin	Polyphenol extracted from turmeric (*Curcuma longa L*.) that downregulates the Nrf2 and NF-κB pathways.	(16,123)
	Kaempferol	A common bioflavonoid present in vegetables and fruits. It reduces iNOS and COX-2 and restrains NF-κB.	(124)
	Apigenin	A naturally occurring plant flavone found in witch-hazel, grapefruit, and other plant sources. It inhibits the NF-κB/Snail pathway.	(125)
	Genistein	An isoflavone present in leguminosae, mainly obtained from the pea family, such as soy beans, fava beans, and even coffee beans. It activates the mTOR pathway and inhibits NF-κB, JNK, and ERK signaling pathways.	(126)

The list of senolytic and senomorphic molecules is extensive; here, we show some of the most commonly used molecules in senotherapy.

The molecular mechanisms of different senotherapeutics are unlikely to depend on a single signaling pathway but rather on the coordinated regulation of multiple nutrient- and stress-sensing networks. Among these, the mechanistic target of rapamycin (mTOR) pathway has emerged as a central integrator of nutrient availability, cellular metabolism, autophagy, inflammatory signaling, and cellular senescence, thereby providing a plausible mechanistic link between dietary bioactive compounds and healthy brain aging. mTOR functions primarily through two complexes, mTORC1 and mTORC2, with mTORC1 integrating signals from amino acids, glucose availability, insulin/IGF-1 signaling, cellular energy status, and oxidative stress to coordinate cellular metabolism, growth, and survival (54, 55). Under nutrient-rich conditions, mTORC1 promotes anabolic metabolism, protein synthesis, and ribosome biogenesis while suppressing autophagy, whereas nutrient deprivation, dietary restriction, or AMPK activation inhibit mTORC1, thereby enhancing autophagic flux, lysosomal function, mitochondrial quality control, and cellular stress resistance (56). These adaptive responses are particularly relevant to aging and neurodegenerative diseases, where impaired autophagy, mitochondrial dysfunction, chronic neuroinflammation, and the accumulation of senescent cells are closely interconnected. Recent evidence further indicates that mTOR signaling regulates transcriptional and epigenetic programs involved in cellular adaptation through direct nuclear functions, linking nutrient sensing with chromatin remodeling and long-term metabolic reprogramming (55). In addition, amino acid-dependent activation of the Rab1A–mTORC1 axis regulates downstream transcriptional programs, illustrating how nutrient sensing can directly influence cellular metabolism and tissue homeostasis (54, 57). Beyond metabolic regulation, mTOR also plays an important role in immune modulation and inflammatory responses by influencing cytokine production, immune cell activation, and the SASP, suggesting that dysregulated mTOR signaling may contribute to inflammaging and age-related neurodegeneration. Consequently, modulation of mTOR activity may represent one of the principal mechanisms through which nutritional senotherapeutics exert senomorphic effects by restoring autophagy, reducing oxidative stress and neuroinflammation, and improving cellular resilience. Nevertheless, because mTOR signaling is highly context-dependent, both chronic hyperactivation and excessive inhibition may produce detrimental effects, highlighting the need for therapeutic strategies aimed at restoring physiological mTOR activity rather than indiscriminate pathway suppression (54–56).

### 3.1 Nutritional senotherapeutics

Dietary ingredients derived from natural products have demonstrated beneficial effects in the prevention and treatment of age-related conditions. These compounds reduce the senescent cell burden and exert anti-inflammatory effects *in vitro*, as and in animal models and humans, highlighting their potential utility as senotherapeutic agents (58).

#### 3.1.1 Nutritional senolytics

##### 3.1.1.1 Quercetin

Quercetin, a flavonoid abundantly found in several plant sources such as onions, apples, grapes, berries, and broccoli, has been widely studied for its neuroprotective potential in AD (59). In a 2019 study by Zhang et al., senolytic treatment with quercetin combined with dasatinib in AD mouse models selectively eliminated senescent cells within the amyloid plaque microenvironment, leading to reduced neuroinflammation, decreased Aβ burden, and improved cognitive performance. These findings support a contributory role of A*β*-induced oligodendrocyte progenitor cell (OPC) senescence in driving neuroinflammatory responses and cognitive impairment in AD, and highlight the therapeutic potential of senolytic interventions (23).

Evidence suggests that it may reduce amyloid-*β* (Aβ) production by modulating the β-secretase activity and attenuate Aβ-induced oxidative stress via its antioxidant properties. Beyond these effects, quercetin exerts neuroprotection through multiple mechanisms, including enhancement of BDNF expression, preservation of mitochondrial integrity, and regulation of apoptotic signaling pathways involving Bcl-2, Bax, cytochrome c, and caspase-3. It also activates the PI3K/Akt pathway and suppresses NF-κB–mediated neuroinflammation (60).

Therefore, quercetin is a promising therapeutic candidate for PD due to its antioxidant, anti-inflammatory, and anti-apoptotic properties. Quercetin modulates multiple molecular pathways implicated in PD pathogenesis, including oxidative stress, apoptosis, autophagy, and neuroinflammatory signaling. These effects are associated with attenuation of tremor and improvements in locomotor performance, motor coordination, and grip strength, likely mediated by the preservation and enhancement of dopamine levels, ultimately resulting in improved behavioral outcomes in experimental PD models (61). Quercetin attenuates MPTP-induced dopaminergic neuronal death by activating nuclear factor erythroid 2–related factor 2 (Nrf2). Activation of Nrf2 promotes the transcription of antioxidant response element (ARE)-driven genes, thereby enhancing the expression of endogenous antioxidant enzymes and mitigating oxidative stress.

Additionally, quercetin modulates the expression of proteins involved in iron uptake and homeostasis, thereby regulating iron-mediated oxidative damage (62). Moreover, quercetin modulates the Janus kinase (JAK)–signal transducer and activator of transcription (STAT) signaling pathway, which mediates the transmission of extracellular signals from membrane receptors to the nucleus, thereby regulating gene expression. Dysregulation of this pathway is associated with neuronal degeneration and demyelination (63).

However, quercetin is among the most extensively investigated dietary flavonoids for neurodegenerative diseases, and the current evidence supporting its role as a nutritional senotherapeutic remains largely preclinical. Different studies performed in transgenic and toxin-induced models of Alzheimer’s and Parkinson’s diseases demonstrate reductions in oxidative stress, neuroinflammation, protein aggregation, and behavioral deficits, supporting the biological plausibility of quercetin as a multitarget neuroprotective compound (59, 61). However, considerable heterogeneity remains across experimental models regarding disease induction, dosage regimens, treatment duration, administration routes, and outcome measures, making direct comparisons between studies challenging and limiting the extrapolation of these findings to clinical settings (61). Future investigations should prioritize standardized experimental protocols, optimization of pharmacokinetic properties, and well-designed clinical trials to determine whether the promising preclinical effects of quercetin can translate into meaningful therapeutic benefits in patients with neurodegenerative diseases.

##### 3.1.1.2 Fisetin

Fisetin is a flavonoid present in various fruits, vegetables, and teas. It is widely used as a dietary supplement due to its diverse pharmacological properties, including antioxidant, anti-inflammatory, antidiabetic, anticancer, antimicrobial, and neuroprotective effects (64). At the molecular level, fisetin modulates key signaling pathways involved in cellular senescence, such as BCL-2, p53, and PI3K/AKT pathway. Its senolytic activity was first identified through a flavonoid screen in senescent human and murine fibroblasts (65, 66). Subsequent studies demonstrated cell–type–specific effects, with reductions in senescent mesenchymal stem/progenitor, immune, and endothelial cells in white adipose tissue (66).

In the context of AD, fisetin modulates inflammatory pathways, preserves cognitive function in transgenic AD models, and enhances memory and hippocampal long-term potentiation in rodents, suggesting that it may increase blood–brain barrier permeability and exert beneficial effects on hippocampal function (67, 68). Collectively, these findings support fisetin as a promising senotherapeutic and neuroprotective candidate in age-related neurodegeneration.

Emerging evidence indicates that fisetin can modulate the phosphoinositide 3-kinase (PI3K)/Akt/mTOR signaling pathway in experimental models of PD. By down-regulating PI3K/Akt/mTOR signaling, fisetin appears to restore autophagic flux, reduce oxidative stress, and protect against dopaminergic neuronal loss (69).

Although these nutritional compounds share the common ability to modulate cellular senescence and neurodegeneration, their mechanisms of action, molecular targets, and translational readiness differ considerably. As summarized in Table 3, quercetin and fisetin have primarily been characterized as senolytic agents, whereas sulforaphane, curcumin, and resveratrol predominantly exhibit senomorphic properties through the modulation of oxidative stress, inflammatory signaling, and metabolic pathways. Likewise, the level of evidence varies substantially among compounds, with robust mechanistic and preclinical support available for most agents, but relatively limited and heterogeneous clinical evidence. Importantly, the comparison also highlights common translational challenges—including poor bioavailability, limited BBB penetration, and the scarcity of well-designed randomized clinical trials—which currently represent the principal barriers to the clinical implementation of nutritional senotherapeutics in neurodegenerative diseases.

**Table 3:** Comparative overview of nutritional senotherapeutics: molecular targets, experimental evidence, and translational considerations in neurodegenerative diseases.

Compound	Senotherapeutic classification	Main molecular targets/pathways	Experimental evidence	Clinical/human evidence	Main translational limitations	References
Fisetin	Natural senolytic flavonoid	BCL-2 family, PI3K/AKT, p53, NF-κB; reduction of p16/p21-associated senescence	Strong senolytic activity in cell and animal models; reduces senescence markers and improves age-related phenotypes in experimental models.	Very limited direct evidence in neurodegenerative diseases; mainly preclinical and early translational evidence.	Scarcity of randomized clinical trials; need for pharmacokinetic and CNS-delivery studies.	(127–129)
Sulforaphane	Natural senomorphic isothiocyanate,.	Nrf2/Keap1, NF-κB, antioxidant response elements, inflammatory signaling.	Strong mechanistic and preclinical evidence in AD/PD-related models reduces oxidative stress, neuroinflammation, amyloid/tau pathology, and dopaminergic damage.	Limited but growing human evidence; biological activity and tolerability reported, but direct evidence in neurodegenerative diseases remains scarce.	Variability in glucoraphanin content, myrosinase activity, gut microbiota conversion, formulation, and dose standardization.	(73, 78, 79)
Curcumin	Natural senomorphic polyphenol.	NF-κB, Nrf2, AMPK, PI3K/AKT, inflammatory and oxidative stress pathways.	Extensive preclinical evidence in AD/PD models; reduces neuroinflammation, oxidative stress, amyloid pathology, tau hyperphosphorylation, and cognitive deficits.	Human evidence is heterogeneous; clinical studies and systematic reviews report mixed cognitive outcomes	Very low oral bioavailability, rapid metabolism, limited BBB penetration; efficacy depends strongly on formulation.	(81, 85, 130)
Resveratrol	Natural senomorphic polyphenol / caloric-restriction mimetic.	SIRT1, AMPK, NF-κB, Nrf2, autophagy-related pathways.	Strong preclinical evidence from AD/PD models indicates modulation of oxidative stress, neuroinflammation, mitochondrial dysfunction, proteostasis, and autophagy.	Relatively stronger clinical evidence than other compounds; trials in AD show acceptable safety and biomarker modulation, but cognitive effects are inconsistent.	Low oral bioavailability, rapid metabolism, interindividual variability, anduncertain clinical efficacy.	(91, 92)
Quercetin	Senolytic (particularly in combination with dasatinib); also exhibits senomorphic properties	Inhibits PI3K/AKT, BCL-2 family proteins, NF-κB, and SASP signaling; activates AMPK and Nrf2 pathways, reducing oxidative stress and inflammation.	Numerous *in vitro* and animal studies have demonstrated reduces senescent cell burden, attenuates SASP, decreases oxidative stress and neuroinflammation, improves mitochondrial function, preserves blood–brain barrier integrity, and enhances cognitive performance in models of aging and neurodegenerative diseases.	Clinical evidence remains limited. Early-phase studies of dasatinib plus quercetin (D + Q) have shown acceptable safety, reduced senescence-associated biomarkers, and improved physical function in patients with diabetic kidney disease and idiopathic pulmonary fibrosis. Preliminary studies in Alzheimer’s disease suggest reductions in senescence-related biomarkers and acceptable CNS penetration, although efficacy on clinical outcomes has not yet been established.	Poor oral bioavailability, lack of standardized dosing regimens, small clinical sample sizes, and insufficient randomized controlled trials evaluating long-term efficacy and safety.	(127–129)

Although experimental studies suggest that fisetin may modulate oxidative stress, neuroinflammation, synaptic dysfunction, and senescence-associated pathways (70–72), the available literature is limited by the heterogeneity of experimental models, differences in dosing regimens and treatment duration, and the scarcity of studies evaluating senescence-specific endpoints in the central nervous system. Therefore, future studies should prioritize standardized preclinical protocols, pharmacokinetic characterization, direct evaluation of senescence biomarkers, and well-designed clinical trials to determine whether fisetin can be translated from a promising experimental senotherapeutic into a clinically useful intervention.

#### 3.1.2 Nutritional senomorphics

##### 3.1.2.1 Sulforaphane

Sulforaphane (SFN), chemically known as 1-isothiocyanato-4-(methylsulfinyl)butane, is an isothiocyanate derived from its precursor glucoraphanin, which is predominantly found in cruciferous vegetables such as broccoli, cauliflower, cabbage, and brussels sprouts. SFN is a phytochemical with well-documented antioxidant, anti-inflammatory, and anti-apoptotic properties. Given that oxidative stress, chronic inflammation, and mitochondrial dysfunction are central mechanisms underlying neurodegenerative diseases, SFN has emerged as a promising candidate for neuroprotection (73).

The neuroprotective effects of sulforaphane have been demonstrated in multiple experimental models of AD. Hou et al. reported that intraperitoneal administration of SFN (5 mg/kg) for 4 months in PS1V97L transgenic mice prevented cognitive decline, as assessed by behavioral testing. SFN treatment reduced Aβ aggregation, tau hyperphosphorylation, oxidative stress markers (increased GSH and decreased malondialdehyde levels), and pro-inflammatory cytokines (TNF-*α* and IL-1β) (74). These findings suggest that sulforaphane may inhibit Aβ oligomer production in AD.

In 3 × Tg-AD mice, oral SFN (10 or 50 mg/kg) improved cognitive performance and significantly decreased Aβ accumulation in the cortex and both Aβ and tau levels in the hippocampus, as revealed by novel object/location recognition tests and contextual fear conditioning tests (75).

Collectively, these studies indicate that SFN mitigates amyloid pathology, tau alterations, oxidative stress, and neuroinflammation, supporting its potential as a multitarget neuroprotective strategy in AD.

In PD, multiple *in vitro* and *in vivo* studies have demonstrated the neuroprotective efficacy of SFN. Given that endogenous Nrf2 activation is often insufficient to prevent progressive neuronal loss, pharmacological induction of the Nrf2 pathway by compounds such as SFN represents a promising strategy to mitigate neurodegeneration and slow disease progression (76). MPTP-exposed male C57BL/6 mice received either glucoraphanin-enriched pellets or a standard control diet for 28 days. The results showed that dietary supplementation with 0.1% glucoraphanin significantly protected dopaminergic neurons from neurodegeneration, as evidenced by preserved tyrosine hydroxylase immunoreactivity (77).

Compared with other polyphenols, sulforaphane and its precursor glucoraphanin have relatively favorable bioavailability; however, their biological effects depend on multiple variables, including the glucoraphanin content of dietary sources, myrosinase activity, gut microbiota metabolism, formulation, and interindividual variability (78, 79). Although preclinical studies in models of Alzheimer’s and Parkinson’s diseases support its ability to reduce oxidative stress, neuroinflammation, amyloid-related pathology, and dopaminergic damage, the translational relevance of these findings remains limited by differences in experimental doses, routes of administration, and the results across studies (73). Thus, despite its strong mechanistic rationale, future studies should determine whether dietary intake alone is sufficient to achieve biologically active concentrations or whether standardized glucoraphanin/sulforaphane formulations are required for reproducible clinical efficacy.

##### 3.1.2.2 Curcumin

Curcumin ((1E,6E)-1,7-bis(4-hydroxy-3-methoxyphenyl)-1,6-heptadiene-3,5-dione) is the principal compound of *Curcuma longa* (*C. longa*), commonly known as turmeric. Turmeric is commonly used as a culinary spice, particularly in Indian cuisine and other South Asian dishes (80).

Curcumin is a potential therapeutic agent in AD pathogenesis. It reduces brain inflammation and protects against A*β*-induced neurotoxicity. In animal models, oral administration of curcumin ameliorates behavioral deficits, reduces amyloid-β (Aβ) deposition, inhibits tau hyperphosphorylation, and attenuates amyloid-associated neurotoxicity. These effects suggest a capacity to modulate key pathological pathways involved in AD progression. Some clinical studies have also reported cognitive benefits following curcumin supplementation; however, evidence in humans remains limited (81). Curcumin exerts its anti-inflammatory effects primarily by suppressing the NF-κB signaling pathway. Specifically, curcumin inhibits activation of the IKK complex, thereby preventing NF-κB nuclear translocation and subsequent transcription of pro-inflammatory genes (82).

In the PD context, curcumin exhibits potent anti-inflammatory, antioxidant, and free–radical–scavenging properties, mediated through multiple molecular and cellular mechanisms. In experimental models, curcumin modulates *α*-synuclein aggregation, a central feature of PD pathology. Furthermore, curcumin may exert therapeutic effects in PD by modulating key neurodegeneration-associated signaling pathways, including PI3K/Akt and BDNF, thereby promoting neuronal survival and functional recovery (83). In a rat model of PD, curcumin supplementation attenuated neuroinflammation by downregulating pro-inflammatory cytokines and NF-κB signaling, while also modulating key mediators of the apoptotic pathway, thereby contributing to neuronal protection (84).

Nevertheless, the therapeutic interpretation requires caution because many studies differ substantially in formulation, dose, route of administration, treatment duration, and disease model. A major translational limitation is curcumin’s poor oral bioavailability, which is related to low aqueous solubility, limited intestinal absorption, rapid metabolism, and systemic elimination (85). Although curcumin-based nanoparticles, liposomes, and other delivery systems have been developed to improve stability, bioavailability, and central nervous system delivery, these approaches remain insufficiently validated in large clinical studies (86). Therefore, while curcumin remains a biologically plausible nutritional senomorphic candidate, stronger clinical evidence is required before its efficacy in neurodegenerative diseases can be established.

##### 3.1.2.3 Resveratrol

Resveratrol is a polyphenolic compound predominantly found in pigmented fruits and vegetables, including grapes, blueberries, and cranberries, as well as in peanuts, cocoa, and wine. Recognized as one of the most extensively studied phytochemicals with potential anti-aging effects, resveratrol has been investigated for its antioxidant, anti-inflammatory, and senescence-modulating properties (87).

Resveratrol significantly reduces cerebrospinal fluid (CSF) matrix metalloproteinase-9 (MMP-9) levels and modulates neuroinflammatory markers in patients with AD compared with placebo-treated controls. These findings suggest that activation of SIRT1 by resveratrol may represent a key mechanism underlying its potential therapeutic or preventive effects in neurodegenerative disorders. Clinical data further indicate that resveratrol is generally well tolerated in patients with AD (88).

In the case of PD, resveratrol treatment significantly protected mice against MPTP-induced oxidative stress, motor coordination impairment, and neuronal death. Likewise, resveratrol attenuated 6-hydroxydopamine-induced oxidative injury and dopamine depletion in a rat model of PD.

Beyond its systemic effects, resveratrol also modulates astroglial function. Its neuroprotective actions in glial cells involve regulation of glutamatergic homeostasis following synaptic glutamate release. Specifically, resveratrol enhances astrocytic glutamate uptake, promotes glutamine synthetase activity—facilitating the conversion of glutamate to glutamine—and stimulates glutathione synthesis, thereby strengthening antioxidant defenses and reducing excitotoxic stress (89).

Experimental data consistently demonstrate that resveratrol modulates multiple hallmarks of brain aging, including oxidative stress, mitochondrial dysfunction, neuroinflammation, proteostasis impairment, and cellular senescence through activation of SIRT1/AMPK signaling and inhibition of NF-κB-dependent inflammatory pathways (88, 90). Clinical studies further suggest that resveratrol is generally well tolerated and may reduce neuroinflammatory biomarkers, including cerebrospinal fluid MMP-9, while preserving BBB integrity; however, improvements in cognitive performance have been modest and inconsistent across trials (91, 92). As with other polyphenols, its clinical translation remains limited by low oral bioavailability, rapid metabolism, and interindividual variability in systemic exposure.

Despite their promising senotherapeutic properties in experimental models, the clinical translation of dietary bioactive compounds remains limited by several pharmacokinetic challenges. Curcumin, resveratrol, and quercetin exhibit low oral bioavailability due to poor aqueous solubility, extensive first-pass metabolism, and rapid systemic elimination, which substantially reduce their circulating concentrations and tissue distribution (8, 93). Furthermore, although these compounds have demonstrated the ability to cross the BBB in experimental studies, their brain penetration is generally limited and may not consistently achieve therapeutically effective concentrations under physiological conditions (90). These limitations have prompted the development of alternative delivery strategies, including nanoformulations, lipid-based carriers, and structural modifications designed to improve bioavailability, brain delivery, and therapeutic efficacy. Therefore, while nutritional senotherapeutics remain promising candidates for promoting healthy brain aging, overcoming these pharmacological barriers will be essential for their successful clinical translation.

Nutritional senotherapeutics represent promising strategies for mitigating age-related diseases and promoting healthspan; these compounds are present in several foods (fruits, vegetables, seeds, etc.) and offer an alternative to pharmaceutical drugs and their long-term adverse effects. However, important translational challenges are shared by most nutritional senotherapeutics, including limited oral bioavailability, variable BBB penetration, lack of standardized formulations and dosing regimens, substantial interindividual variability in metabolism, and the scarcity of adequately powered randomized clinical trials (14, 58). Another important limitation is the lack of standardized biomarkers that enable reliable assessment of the senescence burden and therapeutic response in humans, making it difficult to compare outcomes across studies and to establish clinically meaningful endpoints.

## 4 Clinical evidence and translational perspective of nutritional senotherapeutics

Current evidence supporting nutritional senotherapeutics follows a translational continuum from mechanistic studies to early clinical evaluation. Initial *in vitro* studies demonstrated that quercetin attenuates several hallmarks of cellular senescence by reducing senescence-associated *β*-galactosidase (SA-β-gal) activity and modulating NF-κB/SIRT1 signaling (94), whereas fisetin decreases p16- and p21-associated senescence and selectively eliminates senescent cells while preserving normal cell viability (66). Likewise, sulforaphane activates the Nrf2 antioxidant pathway, thereby reducing oxidative stress and inflammatory signaling in astrocytes and neuronal cells, while resveratrol suppresses SASP-associated inflammatory responses by activating SIRT1 and inhibiting NF-κB signaling (73). These mechanistic findings have been consistently validated in experimental animal models. In an Alzheimer’s disease mouse model, senolytic treatment with dasatinib plus quercetin reduced the number of senescent oligodendrocyte progenitor cells, attenuated neuroinflammation, decreased amyloid burden, and improved cognitive performance (23). Similarly, sulforaphane ameliorated cognitive impairment and neuropathological alterations through activation of Nrf2-dependent antioxidant pathways (77), curcumin attenuated neuroinflammation and improved learning and memory in transgenic Alzheimer’s disease models (95), whereas resveratrol exerted neuroprotective effects in experimental Parkinson’s disease by promoting SIRT1-mediated autophagy and reducing dopaminergic neuronal loss (96). Collectively, these findings provide compelling mechanistic and preclinical evidence supporting the senotherapeutic potential of dietary bioactive compounds.

## 5 Senotherapy in the CNS: eliminate or modulate?

The homeostasis of senescent cells is fundamental for their physiological functions and healthy aging, as they are known to be beneficial in the short term by participating in processes such as tissue repair, regeneration, and protection against cancer. The maximum benefit of senescence is achieved when its presence is temporally limited by immune clearance, thereby avoiding adverse effects (97). Therefore, it is crucial to determine whether it is better to eliminate them or modulate their secretome to induce a less inflammatory profile.

The timing of senolytic treatment and the adverse effect of senescent cell removal are unresolved issues, as the loss of too many cells could be worse than that of persistent senescent cells, because they have a variety of beneficial physiological functions, and interfering with these functions potentially carries health risks that may restrict the use of senolytics in specific populations. It should be assessed whether the benefits outweigh the risks to overall health (98). It is essential to consider the use of senolytics in organs that cannot regenerate their cells, such as the brain. Adult neurogenesis is not a CNS-wide regenerative process, but rather is restricted to specific regions, such as the olfactory bulb and the dentate gyrus of the hippocampus. Notably, neural precursor cells (NPCs), rather than mature neurons, proliferate and subsequently differentiate into neurons; however, their capacity to restore overall brain function is limited (99). Since neurons do not divide, their elimination by senolytics results in permanent loss; it is crucial to determine if they are affected by senolytics when considering these drugs as CNS interventions. While eliminating senescence-like neurons may be therapeutic in certain conditions (4), it is essential to carefully consider the consequences of removing these largely irreplaceable postmitotic cells, including the timing of their removal to avoid major impairments in brain function and potential drug side effects (50, 100).

**Figure 1:**
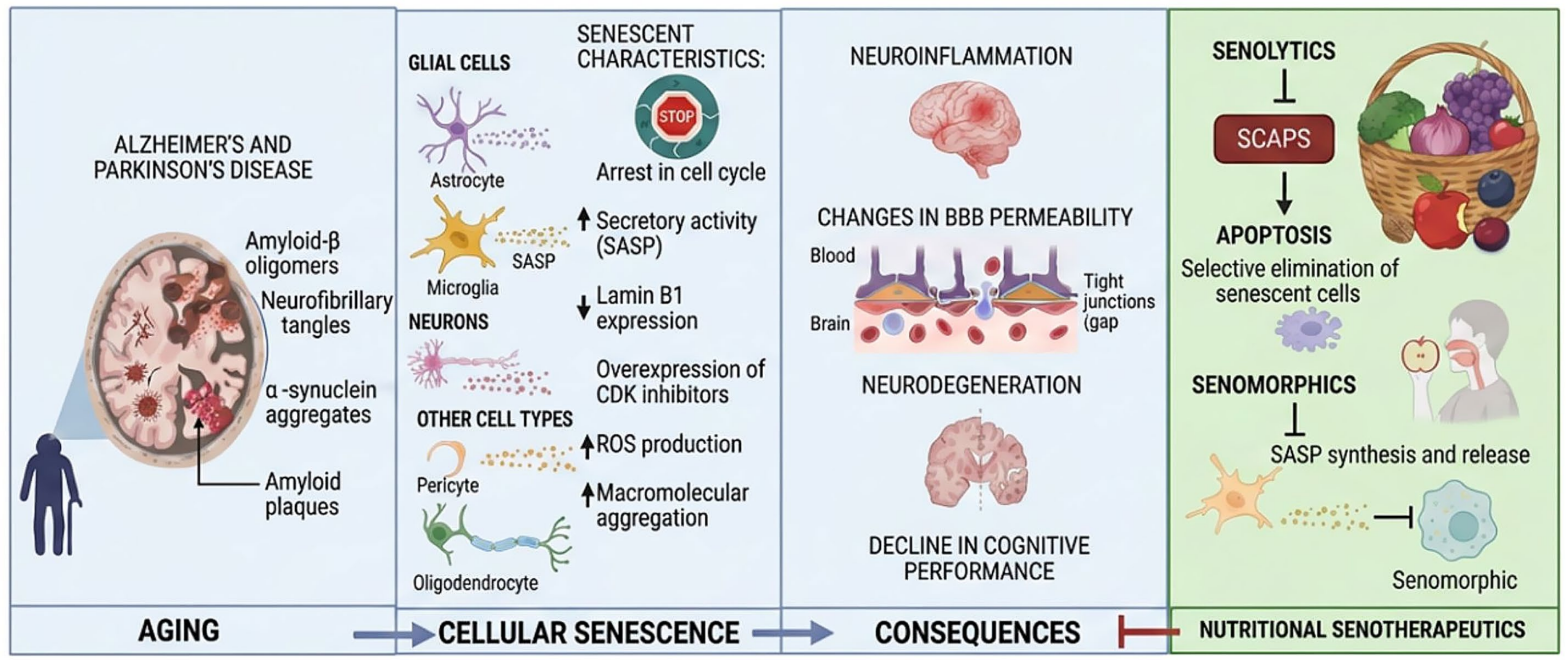
Nutritional senotherapeutics in neurodegenerative diseases. Aging drives the accumulation of senescent cells in the brain, including astrocytes, microglia, neurons, and oligodendrocytes, which are characterized by the SASP, oxidative stress, and protein aggregation. These senescent cells contribute to neuroinflammation, blood–brain barrier dysfunction, and neurodegeneration, which are central to AD and PD. The figure also highlights nutritional senotherapeutics derived from dietary intake as an emerging strategy, with senolytics selectively eliminating senescent cells and senomorphics attenuating the harmful components of the SASP.

In the case of senomorphics, SASP modulation is also a consideration, as SASP components promote inflammation and tissue damage; however, certain elements support tissue regeneration, immune surveillance, and fibrosis resolution (52). Consequently, future senomorphic approaches based on dietary intake should focus on selectively modulating the SASP, suppressing harmful secretions while preserving or enhancing its beneficial effects (Figure 1).

To ensure the effectiveness, safety, and reproducibility of senotherapy, several key aspects must be addressed. First, it is essential to define the optimal therapeutic window for intervention, including the patient’s age at the time of treatment. Second, the organism’s health status must be carefully evaluated, as some pathologies can alter the drug’s bioavailability at the site of action. Finally, the most appropriate dosing schedule—whether continuous, periodic, or intermittent—should be established, alongside the identification of reliable biomarkers that accurately reflect therapeutic efficacy.

Although nutritional senotherapeutics are generally considered to have favorable safety profiles when consumed as part of a balanced diet or within clinically evaluated supplemental doses, their widespread use as dietary supplements warrants careful consideration, particularly among older adults with multiple comorbidities. Several bioactive compounds may interact with commonly prescribed medications (8). Moreover, the variability in supplement formulations, differences in bioavailability, and the limited long-term safety data available for high-dose supplementation underscore the importance of individualized risk–benefit assessment and medical supervision before recommending these compounds as senotherapeutic interventions in clinical practice.

Although senotherapeutic strategies have shown considerable promise in mitigating brain aging and neurodegeneration, their clinical translation in its early stages. Most evidence derives from preclinical studies, and important questions regarding optimal timing, treatment duration, target cell populations, and long-term safety remain unresolved. Furthermore, the dual role of cellular senescence in tissue homeostasis and repair highlights the need for caution when considering senescent cell elimination as a therapeutic strategy. In this context, nutritional senotherapeutics should be viewed as complementary interventions that modulate senescence-associated pathways rather than as replacements for conventional therapies. Their successful clinical translation will require improved bioavailability, reliable biomarkers of senescence, and well-designed clinical trials to establish their efficacy and safety in patients with neurodegenerative diseases.

## 6 Blood–brain barrier permeability and bioavailability: major challenges for the clinical translation of nutritional senotherapeutics

The BBB remains one of the principal obstacles to the successful clinical translation of senotherapeutic strategies for neurodegenerative diseases. As a highly selective interface, the BBB tightly regulates the transport of endogenous molecules and therapeutic agents into the central nervous system, limiting the entry of approximately 98% of small-molecule drugs and nearly all biological therapeutics (101–103). Furthermore, BBB permeability is dynamically influenced by aging, neurodegeneration, systemic inflammation, metabolic status, and diet, all of which can alter drug penetration and therapeutic efficacy (104).

These challenges are particularly relevant for nutritional senotherapeutics. As summarized in Table 3, although quercetin, fisetin, sulforaphane, curcumin, and resveratrol have demonstrated robust senescence-modulating and neuroprotective effects in experimental models, most are characterized by poor oral bioavailability, rapid metabolism, and variable BBB permeability, limiting their clinical translation (58, 59). Consequently, advanced delivery strategies (including nanoparticles, liposomes, phytosomes, and other nanocarrier-based systems) have emerged as promising approaches to enhance compound stability, brain delivery, and therapeutic efficacy (105). Nevertheless, clinical evidence supporting the superiority of these formulations remains limited, underscoring the need for further pharmacokinetic optimization and well-designed translational studies.

## 7 Conclusion and perspectives

An excessive and persistent accumulation of senescent cells is a hallmark of aging. This buildup contributes to tissue dysfunction, including in the central nervous system. Here, cellular senescence has been mechanistically linked to the pathogenesis of Alzheimer’s and Parkinson’s diseases. Preclinical evidence supports the concept that targeting senescent cells can ameliorate pathological features.

Natural compounds such as quercetin, fisetin, and sulforaphane have demonstrated senotherapeutic activity *in vitro* and in animal models. They act either by modulating the SASP or by promoting the selective elimination of senescent cells. However, although dietary senotherapeutics represent a biologically plausible and attractive strategy, current clinical evidence remains limited. It is not yet sufficient to determine whether their intake meaningfully reduces senescent cell burden in the human brain or translates into measurable clinical benefits; this precludes formal therapeutic recommendations at this stage.

An additional challenge for the clinical translation of nutritional senotherapeutics is determining whether the concentrations shown to exert senotherapeutic effects in experimental models can be achieved through dietary intake alone. In most preclinical studies, bioactive compounds are administered at doses substantially higher than those typically obtained from habitual diets. Consequently, human clinical studies have generally relied on standardized dietary supplements rather than food sources to achieve potentially therapeutic concentrations. However, considerable variability in absorption, metabolism, and individual responses, together with the lack of standardized dosing regimens, currently precludes the establishment of optimal intake recommendations for neuroprotection or senotherapeutic applications.

Moreover, despite the growing body of preclinical evidence supporting the senotherapeutic potential of dietary bioactive compounds, several challenges must be addressed before these interventions can be translated into routine clinical practice. Future research should prioritize identifying reliable, standardized biomarkers of cellular senescence, optimizing formulations to improve bioavailability and BBB penetration, and implementing adequately powered randomized clinical trials that incorporate clinically meaningful neurological outcomes. Furthermore, emerging approaches such as nanotechnology-based drug delivery systems, multi-omics integration, and precision geroscience are expected to enhance the development of personalized senotherapeutic strategies tailored to the biological heterogeneity of neurodegenerative diseases. Rather than replacing conventional pharmacological therapies, nutritional senotherapeutics are better positioned as complementary interventions within multimodal strategies to promote healthy brain aging and delay the onset or progression of neurodegenerative disorders.

Collectively, these considerations underscore both the promise and the current limitations of senotherapeutic strategies for neurodegenerative diseases. While preclinical studies provide compelling mechanistic evidence for modulating cellular senescence, significant translational challenges remain, including identifying reliable biomarkers, optimizing therapeutic delivery, and validating in well-designed clinical trials. Addressing these challenges will be essential for translating senotherapeutic approaches from experimental models to clinical practice.
